# stImage: a versatile framework for optimizing spatial transcriptomic analysis through customizable deep histology and location informed integration

**DOI:** 10.1093/bib/bbaf429

**Published:** 2025-09-04

**Authors:** Yu Wang, Haichun Yang, Ruining Deng, Yuankai Huo, Qi Liu, Yu Shyr, Shilin Zhao

**Affiliations:** Department of Biostatistics, Vanderbilt University Medical Center, 2525 West End Avenue, Suite 1100, Nashville, TN 37232, United States; Center for Quantitative Sciences, Vanderbilt University Medical Center, 2525 West End Avenue, Suite 1020, Nashville, TN 37232, United States; Department of Pathology, Microbiology and Immunology, Vanderbilt University Medical Center, 1161 Medical Center Dr, Nashville, TN 37240, United States; Department of Computer Science, Vanderbilt University, Sony Bmg, 1400 18th Ave S, Nashville, TN 37212, United States; Department of Computer Science, Vanderbilt University, Sony Bmg, 1400 18th Ave S, Nashville, TN 37212, United States; Department of Biostatistics, Vanderbilt University Medical Center, 2525 West End Avenue, Suite 1100, Nashville, TN 37232, United States; Center for Quantitative Sciences, Vanderbilt University Medical Center, 2525 West End Avenue, Suite 1020, Nashville, TN 37232, United States; Department of Biostatistics, Vanderbilt University Medical Center, 2525 West End Avenue, Suite 1100, Nashville, TN 37232, United States; Center for Quantitative Sciences, Vanderbilt University Medical Center, 2525 West End Avenue, Suite 1020, Nashville, TN 37232, United States; Department of Biostatistics, Vanderbilt University Medical Center, 2525 West End Avenue, Suite 1100, Nashville, TN 37232, United States; Center for Quantitative Sciences, Vanderbilt University Medical Center, 2525 West End Avenue, Suite 1020, Nashville, TN 37232, United States

**Keywords:** optimizing, spatial transcriptomics, integration, histology images, deep learning

## Abstract

Spatial transcriptomics (ST) integrates gene expression data with the spatial organization of cells and their associated histology, offering unprecedented insights into tissue biology. While existing methods incorporate either location-based or histology-informed information, none fully synergize gene expression, histological features, and precise spatial coordinates within a unified framework. Moreover, these methods often exhibit inconsistent performance across diverse datasets and conditions. Here, we introduce stImage, an open-source R package that provides a comprehensive and flexible solution for ST analysis. By generating deep learning–derived histology features and offering 54 integrative strategies, stImage seamlessly combines transcriptional profiles, histology images, and spatial information. We demonstrate stImage’s effectiveness across multiple datasets, underscoring its ability to guide users toward the most suitable integration strategy using diagnostic graph. Our results highlight how stImage can optimize ST, consistently improving biological insights and advancing our understanding of tissue architecture. stImage is freely available at https://github.com/YuWang-VUMC/stImage.

## Introduction

Spatial transcriptomics (ST) measures transcriptional profiles in their spatial context along with corresponding histological images [1–3] providing a multidimensional view of cellular organization. This approach presents an unprecedented opportunity to characterize spatial expression heterogeneity [2]. Fully exploiting multiple data modalities holds great promise for gaining a deep understanding of cellular structures and microenvironment [3], which is crucial for uncovering mechanisms of disease and developing novel therapeutic strategies.

Several innovative methods have been developed to leverage this wealth of information, successfully defining spatial structures that are missed by single modality approaches [4–16]. Some methods employ location-informed strategies. For instance, GraphST [15] and SpaGCN [7] utilize graph convolutional networks to derive spot representations by minimizing the embedding distances between spatially adjacent spots. SpatialPCA [11] models the spatial correlation structure across tissue locations during dimensionality reduction. BASS [16] and BayesSpace [14] leverage spatial neighborhood information through Bayesian statistical strategies for resolution enhancement or clustering analysis. Other approaches combine histology images and/or spatial location with expression profiles [4, 7, 8]. MUSE [4], for example, learns joint latent features by integrating morphological image features and transcriptomic data using a self-supervised autoencoder. Although these innovative approaches have greatly contributed to the advancement of ST analysis, they primarily focus on gene expression data with the image modality playing a secondary role. More importantly, their effectiveness is highly dependent on assumptions about transcriptomics, spatial, and image patterns. The spatial-aware methods hinge on the assumption of spatially homogenous samples and strong spatial-functional relationships, while methods integrating histology images rely on the quality and the informativeness of the images. Unfortunately, these requirements are not always met, rendering a universal, one-size-fits-all solution elusive.

Recognizing these challenges, we developed a comprehensive and flexible framework, stImage, to optimize ST analysis across different scenarios. stImage not only includes a variety of existing ST analysis approaches [11, 14], but also incorporates many multi-modal integration methods, such as weighted nearest neighbor (WNN) [17] and Spectrum [18]. Equipped with 54 integrative strategies (Supplementary Table S1), stImage enables users to apply and compare different methods simultaneously, as well as make data-driven decisions using its diagnostic graphs that reflect the spatial and histological characteristics inherent in the data.

## Results

### Overview of stImage

**Figure 1:**
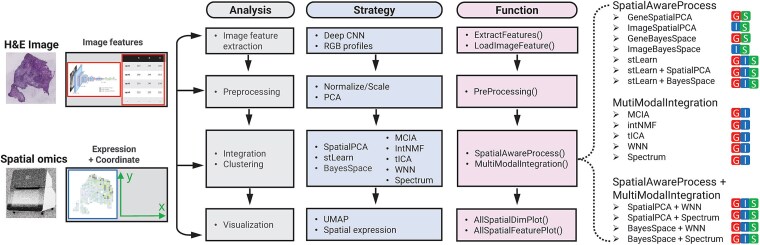
Workflow of stImage. stImage comprises four main steps, image features extraction, preprocessing, data integration, and visualization. The different color squares with abbreviation of different modalities show what combination of modalities were processed for that strategy: G for gene expression; S for spatial coordinates; I for image features.

stImage consists of four main steps (Fig. 1). The first step is to extract image features, either from pre-trained convolutional neural networks (CNN) architectures, or directly from the RGB profile (Details in Methods). The second step is to preprocess the gene expression or image features modalities, respectively. The third step is to integrate three data modalities. stImage provides three ways for integration. One way, named SpatialAwareProcess, focuses on one modality (gene expression or image) and refines it by incorporating spatial and/or image information. stImage equips three spatial-aware approaches, SpatialPCA [11], BayesSpace [14], and stLearn [8]. SpatialPCA and BayesSpace refine gene expression or image features by incorporating spatial location. If gene expression is chosen to be the main modality, the strategy is named GeneSpatialPCA or GeneBayesSpace (Fig. 1). Otherwise, it is called ImageSpatialPCA or ImageBayesSpace. In comparison, stLearn works only on gene expression, adjusting by neighborhood smoothing and morphological similarity. Different SpatialAwareProcess approaches can also be combined. For example, stLearn can be followed by SpatialPCA, resulting in a strategy referred to as “stLearn+SpatialPCA” (Fig. 1). The second way is to treat gene expression and image as two modalities and integrates them by modality integration, including MCIA [20], intNMF [23], tICA [19], Spectrum [18], and WNN [17]. The third way combines SpatialAwareProcess and modality integration. For example, SpatialPCA can be followed by WNN—gene expression and image are first refined separately by SpatialPCA and then integrated by WNN. Figure 1 listed 16 commonly used strategies. The wide range of analysis strategies provided by stImage (Supplementary Table S1) offers flexibility in choosing which modalities to integrate and how to integrate them. The final step is visualization using UMAP projections, gene expression maps, or clustering results overlaid on the histology image.

### Application on simulation data

**Figure 2:**
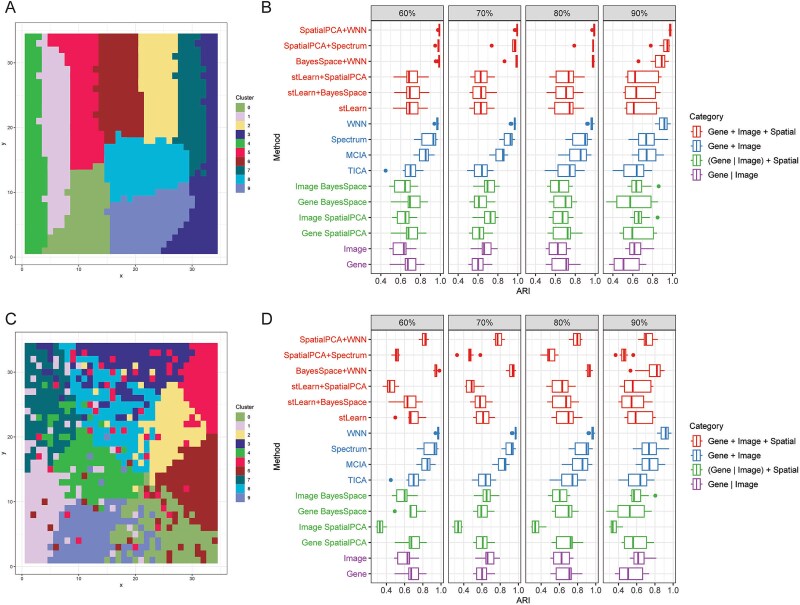
Application of stImage on simulation data with distinct and ambiguous spatial patterns. (A) Illustration of simulation data with a distinct spatial pattern. (B) ARI scores obtained by each strategy on simulation data with distinct spatial patterns and different gene expression dropout rates (60%, 70%, 80%, 90%). (C) Illustration of simulation data with an ambiguous spatial pattern. (D) ARI scores obtained by each strategy on simulation data with ambiguous spatial patterns and different gene expression dropout rates (60%, 70%, 80%, 90%).

We first evaluated the efficiency and applicability of various integrative strategies using simulated ST data with ground-truth cluster labels. The simulated data exhibited either distinct or ambiguous spatial patterns, with varying numbers of clusters (Fig. 2, number of clusters = 10; Supplementary Fig. S1, number of clusters = 4, 6, 8) and different gene expression dropout rates (60%, 70%, 80%, 90%). In the distinct spatial pattern setting, each cluster was exclusively located within specific spatial regions. In contrast, under ambiguous spatial patterns, clusters were not spatially exclusive due to noise (Fig. 2A and C). We utilized the Adjusted Rand Index (ARI) to assess the agreement between ground-truth labels and clusters identified by different strategies (Fig. 2B and D).

As expected, clustering based on gene expression or image alone yielded comparable ARI values (Fig. 2B and D, purple). Integration of gene and image modalities outperformed single-modality strategies (Fig. 2B and D, blue versus purple). Among integration methods, WNN and Spectrum achieved significantly higher ARI scores than MCIA and tICA, which themselves outperformed gene- or image-only methods under high dropout rates. Consistently, the UMAP plots revealed well-separated clusters using WNN and Spectrum (Supplementary Fig. S2). These results suggest that integrating both modalities provides a more accurate characterization of spatial heterogeneity and yields better performance, particularly when both contribute meaningfully to cluster identification.

In simulation data with distinct spatial patterns, SpatialAwareProcess, which refines gene expression or image features using spatial location, showed a slight improvement in ARI (green versus purple in Fig. 2B). Combining SpatialAwareProcess with modality integration, such as SpatialPCAWNN, SpatialPCA+Spectrum, and BayesSpace+WNN, achieved the highest ARI scores. In comparison, although stLearn integrates all three modalities, it performed worse than modality integration alone or the combined “SpatialAwareProcess + modality integration” approaches (Fig. 2B, Supplementary Fig. S3). This suggests that image should be treated as an independent modality, rather than as a local supplement to refine gene expression, particularly when it contributes equally to defining tissue structure. In this context, the optimal integration strategy is to combine all three modalities using SpatialAwareProcess, followed by modal integration.

In simulation data with ambiguous spatial patterns, modality integration approaches that excluded spatial information, especially WNN, outperformed all other strategies. This performance advantage became more pronounced at higher dropout rates (blue in Fig. 2D). When clusters were not spatially homogenous, incorporating spatial information actually compromised the performance (blue versus red in Fig. 2D, Supplementary Fig. S4). In this scenario, the most effective strategy is to integrate gene expression and image data using modality integration while ignoring spatial location.

Additionally, we evaluated the performance of several state-of-the-art ST tools, including Giotto [6] and SpaceFlow [9] on both simulation datasets. As expected, both methods produced results comparable to other spatial-aware approaches in our study, but performed worse than strategies that combined spatial-aware processing with modality integration in the first scenario, and worse than modality integration alone in the second scenario (Supplementary Fig. S5).

In summary, our findings highlight the necessity of choosing suitable integrative strategies that match spatial and image patterns inherent in the data. SpatialAwareProcess improves performance when analyzing data with distinct spatial patterns, whereas methods that omit spatial information perform better on data with ambiguous patterns. When an image alone is informative for defining tissue structures, integrating gene and image modalities improves the performance.

### Application on HER2-positive breast cancer data

**Figure 3:**
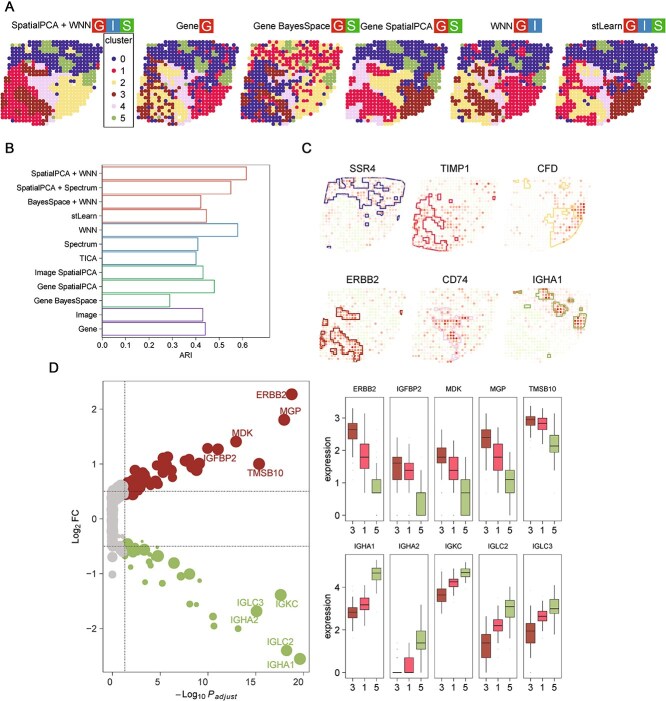
Application of stImage on HER2-positive ST data. (A) Clustering results by six different strategies, SpatialPCA+WNN, gene BayesSpace, gene SpatialPCA, WNN, and stLearn. Cluster legends for the strategy SpatialPCA+WNN: Cluster 0, connective tissue; cluster 1, invasive cancer; cluster 2, adipose tissue; cluster 3, cancer *in situ*; cluster 4, immune infiltrate; cluster 5, breast glands. (B) ARI scores of 12 strategies based on the ground truth labels provided in the original study. (C) Spatial expression of marker genes labeled by the clustering from SpatialPCA+WNN. (D) Differentially expressed genes (DEGs) between the *in situ* cancer region (Cluster 3) and the normal gland region (Cluster 5). Left panel, volcano plot of DEGs, with X-axis showing the negative log_10_ transformed adjusted *P* values (−log_10_ P_adjust_), and Y-axis showing the log_2_ transformed fold changes (log_2_ FC) between *in situ* cancer region and normal gland region. The dot size is positive correlated with the percentage of cells expressing corresponding gene in cluster 3. The top DEGs are labeled. Right panel, boxplot of expression distribution of top DEGs from *in situ* cancer, invasive cancer (cancer surrounding region), or normal gland regions. Y-axis shows the normalized data of genes after SCTransform.

We analyzed HER2-positive breast cancer data of patient H1 generated by the ST platform [24]. 12 of the 54 analysis strategies (Supplementary Table S1) were employed, and their performance was assessed by comparing the agreement between six clusters and six pathologist-annotated regions (Fig. 3).

Strategies using gene expression or image alone both aligned well with pathologists’ annotations (ARI = 0.43–0.44), indicating that both expression and image features contribute to cluster identification (Fig. 3B). This is consistent with the observation that the hematoxylin and eosin (H&E) image clearly delineates the tissue into multiple spatial domains (Supplementary Fig. S6). Among SpatialAwareProcess strategies, SpatialPCA and stLearn improved clustering accuracy (ARI = 0.45–0.48), whereas BayesSpace performed worse (ARI = 0.29). Integration of expression and image modalities using WNN significantly improved the clustering accuracy (ARI = 0.58). However, integration using Spectrum and tICA resulted in lower ARI values (ARI = 0.4–0.41) than single-modality approaches. The higher performance of WNN—which learns the relative importance of each modality for each spot—suggests that the contribution of gene expression and Image features varies across tissue regions. The SpatialPCA followed by WNN strategy achieved the highest ARI value (ARI = 0.62). The ordering of ARI values—Gene/Image < GeneSpatialPCA/ImageSpatialPCA < stLearn < WNN < SpatialPCA+WNN—suggests that both image and spatial information contribute to cluster definition.

In addition to the highest agreement with pathologist annotations, the clusters defined by SpatialPCA+WNN were supported by region-specific genes and pathways. For example, ERBB2 was exclusively expressed in cluster 3, which corresponded to the “in-situ cancer” region in the pathologist’s annotations (Fig. 3C). TIMP1 was highly enriched in cluster 1, which mapped to the “invasive cancer” region. ERBB2 encodes the HER2-receptor, and TIMP1 is associated with breast cancer progression and metastasis [25]. Adipokine CFD [26] was specifically expressed in cluster 2, which aligned with the “adipose tissue” region. Immune response-related gene CD74 was highly enriched in cluster 4, corresponding to the “immune infiltrate” region. Comparing the gene expression between cluster 3 (cancer *in situ*) and 5 (breast glands), we found cancer regions were enriched for growth factor, cell signaling, and tumorigenesis-related pathways. Genes upregulated in cancer regions gradually decreased in expression with increasing distance from the *in situ* cancer region (Fig. 3D).

### Application on pancreatic ductal adenocarcinoma data

**Figure 4:**
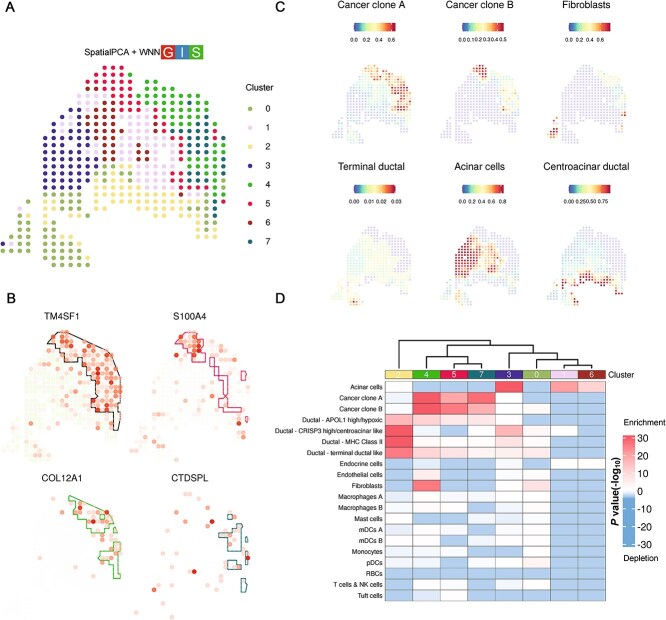
Application of stImage on PDAC-A ST data. (A) Clustering results from the strategy SpatialPCA+WNN. (B) Spatial expression of marker genes in cancer regions and subregions. (C) Cell type composition inferred from deconvolution based on scRNAseq data. Cancer clone A, cancer clone B, and fibroblasts are enriched differently in the three cancer subregions. Acinar cells are enriched in normal pancreatic tissue. Different ductal cells are enriched in different duct epithelium region. (D) Multimodal intersection analysis (MIA) analysis based on genes marking scRNAseq cell types and spatial clustering.

We analyzed a pancreatic ductal adenocarcinoma dataset (PDAC-A) generated by the ST platform [27]. Our findings revealed that strategies using gene expression or image modality alone produced biologically meaningful clustering that aligned well to the overall histological annotation (Supplementary Fig. S7). SpatialAwareProcess approaches produced more spatially contiguous clustering, while combining SpatialAwareProcess with modality integration—particularly SpatialPCA+WNN (Fig. 4A)—yielded the most consistent and interpretable results. The SpatialPCA+WNN clustering was supported by its alignment with four regions defined by the original study and its consistency with cell-type compositions deconvoluted from matched single-cell transcriptomics data [27]. Clusters 4, 5, and 7 identified by SpatialPCA+WNN aligned exclusively with the cancer cells and desmoplasia region. Cluster 2 corresponded to the nonmalignant ductal epithelium region, while cluster 0 lined up with stroma region defined by the original study. Cell type compositions deconvoluted from single-cell transcriptomics data from the same patient further demonstrated that cancer clone A, cancer clone B, and cancer-associated fibroblasts were enriched in cluster 4, 5, and 7, respectively (Fig. 4B and D). Centroacinar ductal cells were exclusively enriched in cluster 2. Notably, SpatialPCA+WNN clustering successfully distinguished cancer clone A (cluster 7) from clone B (cluster 5). Furthermore, the three cancer-associated clusters exhibited different expression patterns. TM4SF1 and S100A4 are known to be highly expressed in malignant cells [28, 29]. TM4SF1 was expressed in all cancer regions, while S100A4 was more specifically expressed in cluster 5. COL12A1 is a collagen encoding gene and highly expressed in cluster 4, marking fibroblast cells as reported in the original paper ([27] Fig. 4A and D). CTDSPL, a known tumor suppressor gene [30], was enriched in cluster 7 (Fig. 4C). In summary, SpatialPCA+WNN, integrating expression, spatial location, and image features, provided a unique and in-depth characterization of the tissue.

### Image-refined transcriptome improves the identification of layer structures of brain

**Figure 5:**
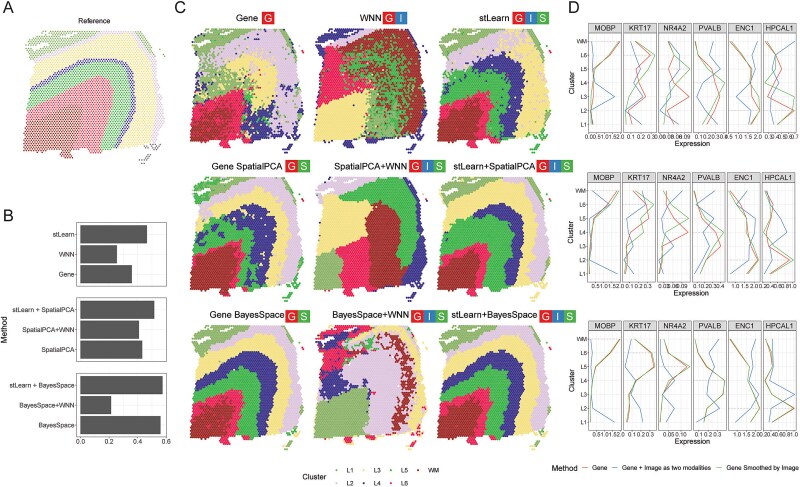
Image-refined transcriptome improves the identification layer structures of brain. (A) Seven layers of human DLPFC; (B) ARI scores obtained by different strategies based on the reference in panel A; (C) clustering results by different strategies. Left: Gene or gene+spatial; middle: Gene + image as two modalities; right, gene expression smoothed by local image; (D) expression pattern of genes marking different layers in clusters defined by different strategies. MOBP (white matter); KRT17 (L5 and L6); NR4A2 (L5); PVALB (L3 and L4); ENC1 (L2); HPCAL1 (L2).

The human dorsolateral prefrontal cortex (DLPFC) exhibits a distinct laminar organization (Fig. 5A), with cells in each layer displaying unique gene expression patterns that differ in morphology, physiology, and connectivity [31]. Using various analysis strategies available in stImage (Supplementary Table S1), we demonstrated that selecting an optimal strategy is essential for accurately decoding the tissue’s complex structure.

Clustering based solely on gene expression produced an ambiguous and noisy layer structure in the DLPFC, with an ARI of 0.36 (Fig. 5B and C). As expected, SpatialPCA and BayesSpace achieved higher ARI values and produced clearer layer structures than clustering based on gene expression alone (GeneSpatialPCA ARI = 0.43; GeneBayesSpace ARI = 0.56), consistent with the spatially distinct architecture of the DLPFC. The H&E image, however, revealed a different pattern (Supplementary Fig. S8A), which did not correspond to the underlying layer structure of DLPFC. As a result, clustering based on image alone yielded a very low ARI value (ARI = 0.18) and failed to reconstruct the underlying tissue structure (Supplementary Fig. S8B). In this case, we found that strategies integrating gene expression and image modalities worsened performance (Fig. 5C; Supplementary Fig. S8B). For example, WNN compared to Gene, SpatialPCA+WNN compared to GeneSpatialPCA, and BayesSpace+WNN compared to GeneBayesSpace all yielded lower ARI scores (Fig. 5B) and disrupted the layer structure (the middle panel in Fig. 5C). In contrast to the reduced performance caused by integrating image data as an independent modality, stLearn—which refines gene expression using local image similarity—slightly improved performance (Fig. 5B and the right panel in the Fig. 5C). The clustering improvements were further supported by the enriched expression of known layer-specific genes, such as MOBP in white matter and L1, KRT17 in L5 and L6, NR4A2 in L5, PVALB in L3 and L4, and ENC1 and HPCAL1 in L2 (Fig. 5D). Clustering analysis across all 12 DLPFC samples yielded similar conclusions (Supplementary Fig. S9).

### Identification of structural layers in the kidney

The kidney has a complex architecture composed of multiple structural layers and intricate components such as the tubules and vessels. Precise identification of its highly organized structures is essential for effectively application of ST analysis to the kidney. We used Visium ST data from mouse kidney to evaluate different methods in stImage and demonstrate the appropriate strategy should be determined based on the characteristics of the sample and data.

**Figure 6:**
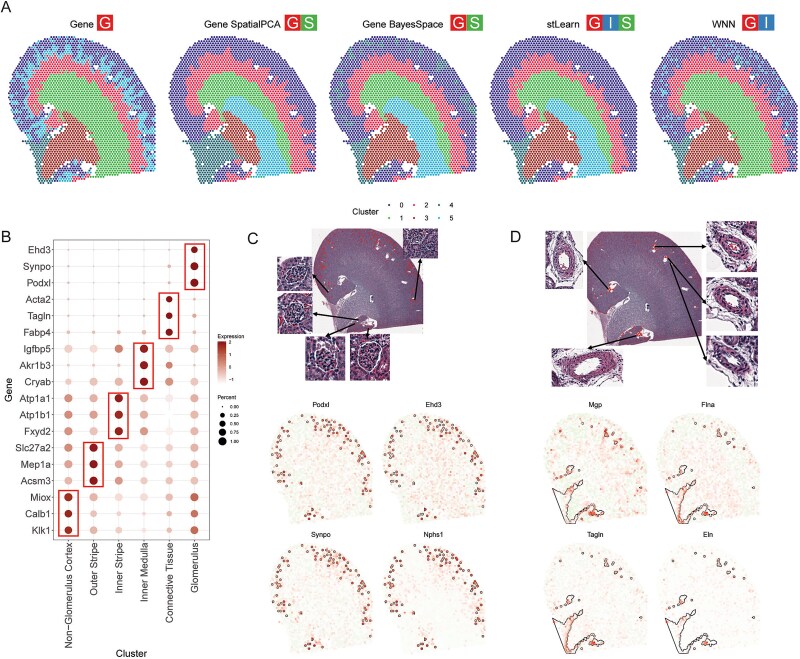
Identification of structural layers in the kidney. (A) Clustering results by gene, gene SpatialPCA, gene BayesSpace, stLearn, and WNN; (B) Dotplot for marker genes for each cluster by the WNN strategy; (C) evaluation on cluster 5 by WNN identified it as a glomerulus cluster (top), and the spatial expression patterns of its marker genes match well with the cluster assignment (bottom); (D) evaluation on spots assigned to connective tissue cluster (cluster 1) by WNN but not by gene expression PCA. They were identified as arteries spots (top) and the spatial expression patterns of their marker genes agree well with the cluster assignment (bottom).

Initially, clustering based solely on gene expression successfully delineated key kidney components including the cortex, outer strip, inner strip, inner medulla, and connective tissue (Fig. 6A). In contrast, spatial-aware techniques, Gene SpatialPCA, Gene BayesSpace, and stLearn, while identifying the major kidney regions, ambiguously subdivided the inner strip. This subdivision was not supported by histological images, and the resulting clusters also lacked distinct marker genes (Supplementary Fig. S10A and S10B). Furthermore, both Gene SpatialPCA and Gene BayesSpace struggled to distinguish kidney tissue from surrounding connective tissue (Fig. 6A). Additionally, SpatialPCA split the inner medulla into two clusters—a division not supported by kidney biology, histological evidence, or the presence of cluster-specific markers (Supplementary Fig. S10C and S10D).

In contrast, the WNN strategy (Fig. 6A, right)—a modality integration method that does not use spatial information—yielded the most accurate results in pinpointing kidney components. It successfully distinguished the cortex, outer strip, inner strip, inner medulla, as well as the connective tissue (Supplementary Fig. S10C and S9D). Notably, the WNN method uniquely identified the glomerulus substructure as a distinct cluster—something not achieved by any other methods (Fig. 6A). In-depth analysis of cluster-specific marker genes (Fig. 6B) further highlighted their correspondence with known kidney structures or connective tissue. For example, the top markers in the Glomerulus cluster included podocyte-specific genes (*Podxl, Synpo, and Nphs1*) or glomerular endothelial cell-specific genes (*Ehd3*), all of which showed spatial expression patterns consistent with the cluster assignment (Fig. 6C). Additionally, the WNN strategy distinguished certain spots within the cortex that were not part of the glomerulus. Subsequent analysis revealed these to be arterial spots within connective tissue, characterized by marker genes predominantly expressed in arteries (*Mgp*, *Flna*, *Tagln, Eln*) [32] (Fig. 6D).

### Prioritizing analysis methods using diagnostic graphs

As demonstrated across these datasets, there is no one-size-fits-all method. To guide strategy selection, we developed three diagnostic plots to help prioritize the use of spatial-aware process and/or modality integration approaches.

**Figure 7:**
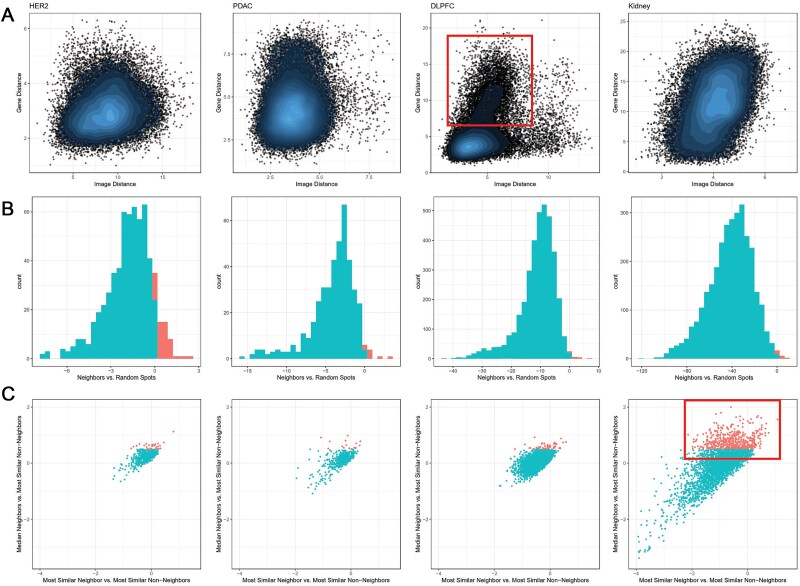
Diagnostic plots for evaluating spatially aware and modality integration assumptions. (A) Scatterplots of Euclidean distances in the gene modality (Y-axis) versus the image modality (X-axis) for each spot pair, computed using the top 20 principal components from each modality. Each point represents a pair of spots. Points in red box suggest modality-specific discrepancies. (B) Histograms comparing the mean similarity of each spot to its spatial neighbors versus randomly selected non-neighbors. Higher neighbor similarity supports the spatially aware processing assumption; conversely, elevated non-neighbor similarity indicates possible violations of that assumption. (C) Scatterplots illustrating spatial heterogeneity by comparing each spot’s similarity to its most similar neighbor relative to its most similar non-neighbor (X-axis) and its average similarity to all neighbors relative to its most similar non-neighbor (Y-axis). Red-marked spots indicate cases where some non-neighbor spots are more similar than neighbor spots, suggesting that spatial proximity alone may not fully capture underlying biological relationships.

Spatial-aware methods assume that each spot is more similar to its neighboring spots than to non-neighboring ones. We first used the Average Similarity Plot to compare each spot’s mean similarity to its neighbors versus a set of randomly chosen non-neighbors (Table 1, Fig. 7B, Supplementary Fig. S11B). In simulation data with distinct spatial patterns, most spots (96%) showed greater similarity to their neighbors (blue bars in Supplementary Fig. S11B). Conversely, in simulations with ambiguous spatial patterns, a notable subset of spots (18%) showed greater similarity to non-neighboring spots (red bars in Supplementary Fig. S11B). In the four real datasets, most spots followed the expected trend of higher similarity to neighboring spots (93%–99%) (Table 1, Fig. 7B). We also used the High-Similarity Non-Neighbor Plot to identify exception spots that were more similar to distant spots than to their neighbors (Table 1, Fig. 7C, Supplementary Fig. S11C). In the simulation data with ambiguous patterns and in the kidney dataset, subset of spots (19% and 16%, respectively) showed stronger similarity to non-neighboring spots (red dots in Fig. 7C and Supplementary Fig. S11C). This observation suggests that relying purely on spatial proximity might overlook meaningful relationships between spots that are not spatially adjacent.

**Table 1:** Results of prioritizing analysis methods using diagnostic graphs. This table summarizes the diagnostic evaluation of different integration strategies using stImage

Data	SpatialAwareProcess		MutiModal integration	Suggested method
	Spot is more similar with its spatial neighbors than non-neighbors?	No spatial non-neighbor more similar than neighbor spots?	Two modalities have consistent patterns	
Simulation, with distinct spatial patterns	Yes (96%)	Yes (97%)	Yes	SpatialAwareProcess + MutiModalIntegration
Simulation, with ambiguous spatial patterns	No (82%)	No (81%)	Yes	MutiModalIntegration
HER2-positive Breast Cancer	Yes (93%)	Yes (95%)	Yes	SpatialAwareProcess + MutiModalIntegration
PDAC	Yes (98%)	Yes (96%)	Yes	SpatialAwareProcess + MutiModalIntegration
DLPFC	Yes (99%)	Yes (99%)	No	SpatialAwareProcess
Kidney	Yes (99%)	No (84%)	Yes	MutiModalIntegration
Mouse Brain (Visium HD)	Yes (93%)	No (74%)	Yes	MutiModalIntegration

Percentages in parentheses indicate the proportion of spots without potential issues (<0.5).

Modality integration methods assume that multiple data modalities exhibit coherent or non-conflicting patterns. To evaluate this, we used Modality Concordance Plot to assess the alignment between gene expression and image-derived features (Fig. 7A and Supplementary Fig. S11A). In most datasets, these plots did not reveal distinct clusters or strong concordance between the two modalities. However, in the DLPFC dataset, a clear pattern emerged in which some spot pairs appeared highly similar in one modality but substantially different in the other. This discrepancy indicates that certain spots may be coherent in one modality yet conflicting in another, underscoring a key challenge for modality integration methods.

A concise summary of these evaluations and the evaluation results was provided in Table 1. Together, these results guided the selection of spatial-aware methods, modality integration approaches, or their combination, depending on the characteristics of each dataset.

## Discussion

In this paper, we introduced stImage, a versatile framework for optimizing ST analysis by integrating gene expression, spatial location, and histological features extracted from deep neural networks. We illustrated the advantages of stImage by customizing integrative strategies to achieve better performance in diverse transcriptomics data. The versatility of stImage was highlighted through its customization potential, enabling the development of the optimal integrative strategy specifically designed for each dataset.

Our results and diagnostic graph indicated the strategy should be guided by the spatial and image pattern inherent in the data, especially focusing on four scenarios as illustrated in Supplementary Fig. S13 and Supplementary Table S1. In the first scenario, when the Modality Concordance Plot shows no discordant patterns and both the Average Similarity Plot and High-Similarity Non-Neighbor Plot reveal only a few spots with potential issues, it indicates image features align well with gene expression, and tissue structures exhibit strong spatial-function coherence. Examples include the simulation data with distinct spatial patterns, HER2-positive breast cancer, and PDAC. In such cases, we recommend applying a spatial aware process followed by modality integration to fully leverage the complementary information. In the second scenario, the Modality Concordance Plot still shows no discordant patterns, but more spots with issues were identified in Average Similarity Plot or/and High-Similarity Non-Neighbor Plot. It suggests that the histological images are informative but spatial heterogeneity in tissue structures becomes prominent, as seen in the simulation data with ambiguous spatial patterns and the kidney study. In these cases, the inclusion of spatial information can even worsen the clustering performance. Therefore, it is preferable to skip the spatial aware process and proceed directly to modality integration. In the third scenario when samples are spatially uniform and histological image is uninformative or conflict with gene expression, such as DLPFC data, skipping the modality integration and only employing a spatially aware process can be advantageous. Finally, when none of the diagnostic plots show meaningful patterns, indicating both uninformative image features and weak or heterogeneous spatial structure. It is advisable to rely solely on gene expression data using standard methods such as PCA.

While multi-modal integration is computationally more intensive and algorithm-dependent, the overall runtime and resource usage of stImage remain manageable even in a typical office desktop, as summarized in Supplementary Table S2. The application of stImage is not limited to spatial transcriptome. We also tested its performance in high-resolution Visium HD data, and the Modality Integration method also had better performance than gene-only (Supplementary Fig. S12). Theoretically, stImage can also be utilized to analyze any spatial omics data, including spatially-resolved proteomics [33] and chromatin accessibility profiling [34]. Moreover, stImage can be extended to combine an increased number of modalities and 3D spatial resolution, aligning with the direction of future spatial omics technologies. Integrative analysis of spatial multi-omics and multi-dimensional data holds great promise for a finer characterization of tissue heterogeneity and enhanced signal from complementary modalities.

In this study, we demonstrated the practicality and versatility of stImage on a wide range of ST datasets across different tissues, platforms, and image resolutions. As we expected, there was no single approach performing well in every dataset. StImage, in contrast, consistently pinpointed the most suitable strategy for each dataset by its diagnostic graph. Thanks to its flexibility and customizability, stImage can take full advantage of multi-views of ST data and capture expression heterogeneity accurately.

## Methods

### Deep learning-based image feature extraction

stImage provides functions to extract deep learning-based image features by different models and parameters via TensorFlow [35] frameworks. Initially, the high-resolution H&E-stained slide is partitioned into small patches based on coordinates from the spatial transcriptomic data. The patch size, defined in pixels, depends on the size of spots with ST data and can also be user-defined. Subsequently, a transfer learning strategy is applied to extract morphological image features from these patches using pre-trained CNN models from ImageNet dataset [36]. stImage utilizes the VGG16 [21] and ResNet50 [22] models, although other models are also available. This transformation converts image data into features that can readily be used for spatial aware data processing and modality integration.

### SpatialAware data processing and modality integration

stImage includes two types of integrative strategies, spatial aware data processing and modality integration. Spatial aware data processing combines spatial location information with expression profiles, while modality integration combines the two independent modalities, expressions, and image, to define latent spaces.

stImage comprises three spatial-aware processing approaches, SpatialPCA, BayesSpace, and stLearn. SpatialPCA is implemented by calling R package SpatialPCA, accessible at (shangll123/SpatialPCA: Spatially aware dimension reduction for spatial transcriptomics. (github.com)) [11]. BayesSpace is implemented by calling R package BayesSpace (Bioconductor - BayesSpace) [14] and the sample similarity matrix was generated based on the results from Markov chain Monte Carlo chain for modality integration by WNN and Spectrum. To incorporate stLearn into stImage, we rewrote its SME_normalize python function with R language.

stImage incorporates five joint dimensional reduction methods for modality integration, intNMF (Integrative non-negative matrix factorization), multiple co-inertia analysis (MCIA), tensorial independent component analysis (tICA), Spectrum, and WNN. The intNMF algorithm is implemented by calling the CRAN R package intNMF (https://cran.r-project.org/web/packages/IntNMF/index.html). MCIA is implemented by calling the R package omicade4 (https://bioconductor.org/packages/release/bioc/html/omicade4.html). tICA is implemented by calling the CRAN R package tensorBSS (CRAN - Package tensorBSS (r-project.org)). Spectrum [18] is implemented by calling the CRAN R package Spectrum (CRAN - Package Spectrum (r-project.org)). WNN is implemented by calling the open-source R package Seurat, accessible at https://www.github.com/satijalab/seurat. Some modifications were made to enable the use of sample similarity matrix as data input.

### Diagnostic plots

stImage provides three types of diagnostic plots to evaluate key assumptions in ST and help guide method selection. All spot-to-spot similarities are based on Euclidean distances computed from the top 20 principal components of each feature space.

To assess whether neighboring spots are more similar than distant ones, we implemented two complementary diagnostics. For each spot, the Average Similarity Plot compares the average similarity to its spatial neighbors versus an equal number of randomly selected non-neighboring spots. The bars extending to the positive side indicate violations of the assumption that neighboring spots are more similar than distant ones. The High-Similarity Non-Neighbor Plot compares a spot’s maximum (X axis) and median (Y axis) similarity to its spatial neighbors against the top 5% of similarities among all non-neighbors. Positive values suggest that some distant spots are more similar than the local neighborhood, highlighting exceptions to spatial locality. A greater number of spots with positive values indicates broader violation of spatial assumptions. These two diagnostics serve complementary purposes: the first captures general spatial coherence, while the second pinpoints specific violations of locality assumptions.

Finally, the Modality Concordance Plot evaluates alignment between different data modalities (gene expression versus image-derived features). We plot pairwise similarities between 5000 randomly sampled spot pairs in each modality. The X-axis shows image-based similarity; the Y-axis shows gene expression-based similarity. Concordance between the two modalities’ similarity matrices indicates consistency and compatibility for multi-modal integration.

### Simulation data

We generated simulated ST data with ground truth cluster labels with two settings, one with distinct spatial patterns and the other with ambiguous spatial patterns. For distinct spatial patterns, we assigned spots to the cluster with the nearest distance to its center. For ambiguous spatial patterns, we assigned cluster labels to a spot based on the joint probability calculated from its distance to each cluster center. In each setting, we generated 16 scenarios with different numbers of clusters (k = 4, 6, 8, 10) and different dropout rates (60%, 70%, 80%, 90%). We repeated each simulation scenario 10 times for performance evaluation.

We generated simulated gene expression and image profiles following the setup in MUSE [4]. Specifically, only a proportion of true cluster identities could be identified from gene expression and image separately, but all clusters could be discriminated against using the combination of both modalities. The gene expression data were generated by a multivariable normal distribution as used in SIMILR [37] and scScope [38]. The same mixture model procedure was used to generate the image modality [4].

### ST datasets

We examined four public ST datasets that include the following.

### HER2 tumor data by ST

The HER2-positive breast tumor data collected by ST platform was downloaded from https://doi.org/10.5281/zenodo.4751624 [24]. Sample H1, which includes 15 029 genes on 613 spots, was examined with stImage. We used the seven spatial domains annotated by pathologists from the original study as ground truth for evaluating. The undetermined regions were excluded when calculating ARIs.

### PDAC-A tumor data by ST

The scRNA-seq and ST data of PDAC-A tumor were both downloaded from the Gene Expression Omnibus (GEO) database with accession number GSE111672.

The PDAC dataset covered 19 738 genes on 428 spots in the pancreatic ductal adenocarcinoma tumor tissue. Preprocessing was conducted to filter out low-expression genes and image features. For single-cell RNAseq (scRNAseq) data, the annotated cell types from the original publication were used for integration analysis and MIA analysis.

### DLPFC human prefrontal cortex data by Visium

We downloaded 12 human DLPFC tissue samples from three individuals on the Visium platform (http://spatial.libd.org/spatialLIBD/). We used sample 151 673 as the main analysis example. The other 11 samples were processed and analyzed with the same methods and parameters, and their results were presented in the Supplementary Figures. Only genes or image features identified in at least 1% of spots were retained.

### Mouse kidney by Visium

The mouse kidney Visium sample data was obtained directly from 10x Genomics Datasets website. The 10x Visium array data contained expressions of 19 465 genes in 3124 spots. Gene and image modalities were processed and normalized, respectively, by stImage package with genes or image features identified in at least 1% of spots retained.

**Key Points**

- **Comprehensive Integration**: *stImage* is the first open-source R package that seamlessly unifies gene expression data, deep learning–derived histological features, and precise spatial coordinates, enabling robust and holistic spatial transcriptomics analyses.
- **Adaptive and Versatile**: Extensive testing across multiple datasets reveals that a one-size-fits-all strategy does not apply. *stImage* offers 54 customizable integration options to accommodate diverse tissue types and experimental conditions.
- **Data-driven Decision Support**: *stImage*’s diagnostic graphs reveal key data characteristics, helping researchers decide when to incorporate histology or spatial data and how to choose the most effective integration strategy for robust and interpretable results.

## Supplementary Material

Supplementary_materials_bbaf429

Supplementary_materials_bbaf429_TableS1

## Data Availability

No new data were generated or analysed in support of this research.
