# Supplementary material for: stImage: a versatile framework for optimizing spatial transcriptomic analysis through customizable deep histology and location informed integration
Source: Brief Bioinform. 2025 Sep 4;26(5):bbaf429. doi: 10.1093/bib/bbaf429 (PMC12409783; doi:10.1093/bib/bbaf429)
Supplement: Supplementary_materials_bbaf429 [file supplementary_materials_bbaf429.docx]

**Supplementary Figure 1:** Adjusted Rand Index of clustering results in simulation data (Number of clusters 4, 6, 8).

A. Number of clusters 4; Simulation data with distinct spatial pattern; B. Number of clusters 4; Simulation data with ambiguous spatial pattern; C. Number of clusters 6; Simulation data with distinct spatial pattern; D. Number of clusters 6; Simulation data with ambiguous spatial pattern; E. Number of clusters 8; Simulation data with distinct spatial pattern; F. Number of clusters 8; Simulation data with ambiguous spatial pattern;


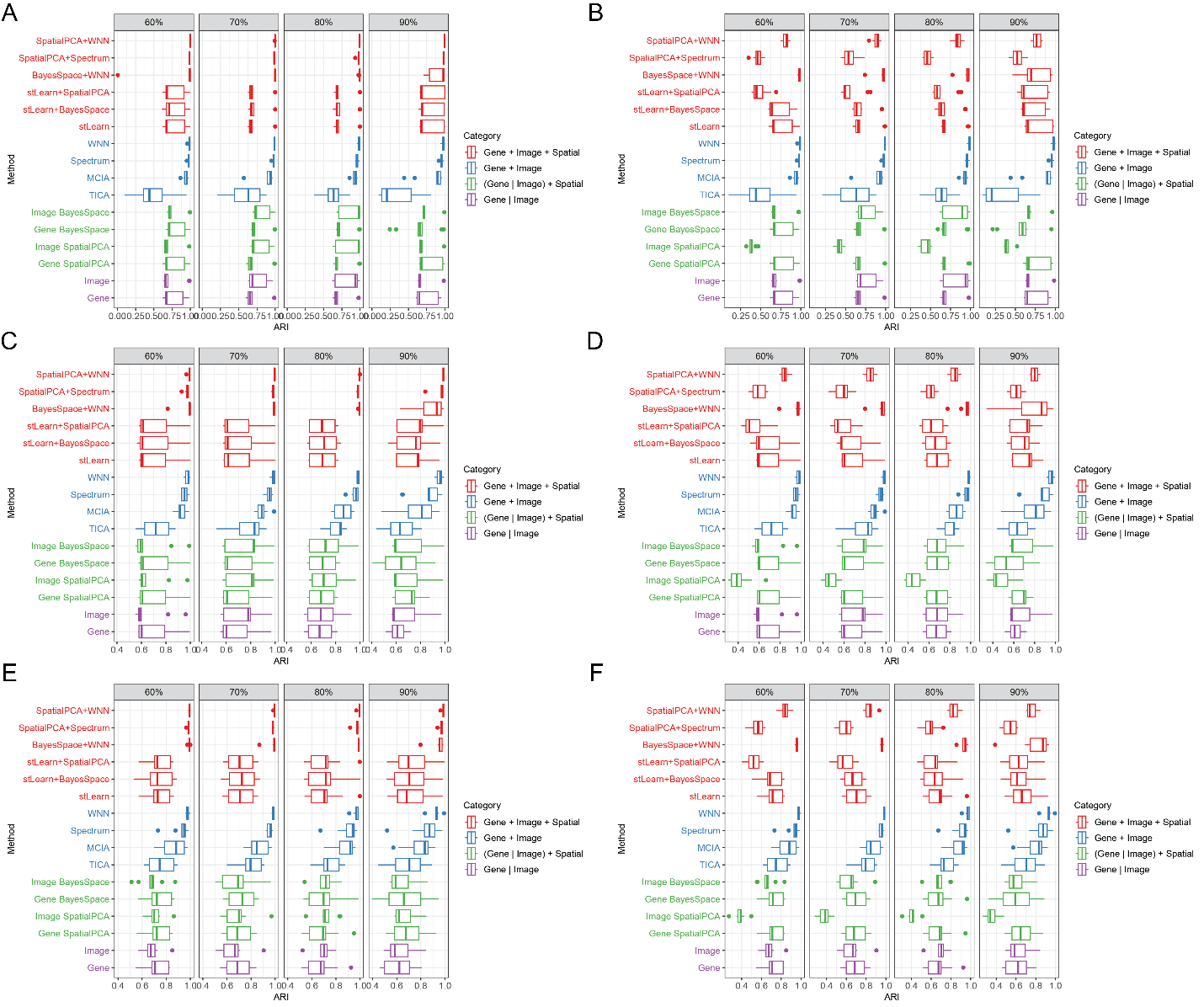


**Supplementary Figure 2:** UMAP of Simulation data with gene and image only.

A. Number of clusters 4; B. Number of clusters 6; C. Number of clusters 8; D. Number of clusters 10;


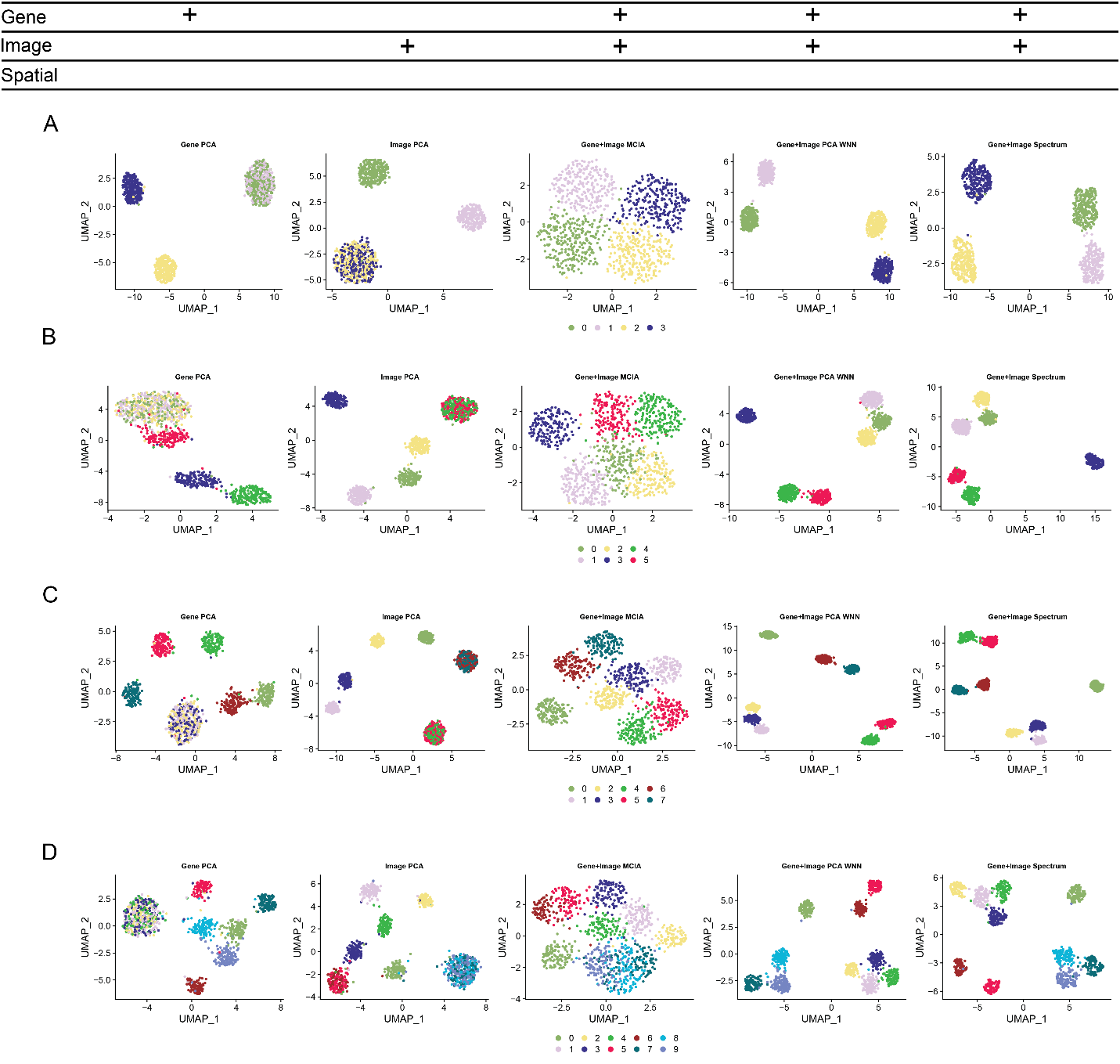


**Supplementary Figure 3:** UMAP of Simulation data with distinct spatial pattern.

A. Number of clusters 4; B. Number of clusters 6; C. Number of clusters 8; D. Number of clusters 10;


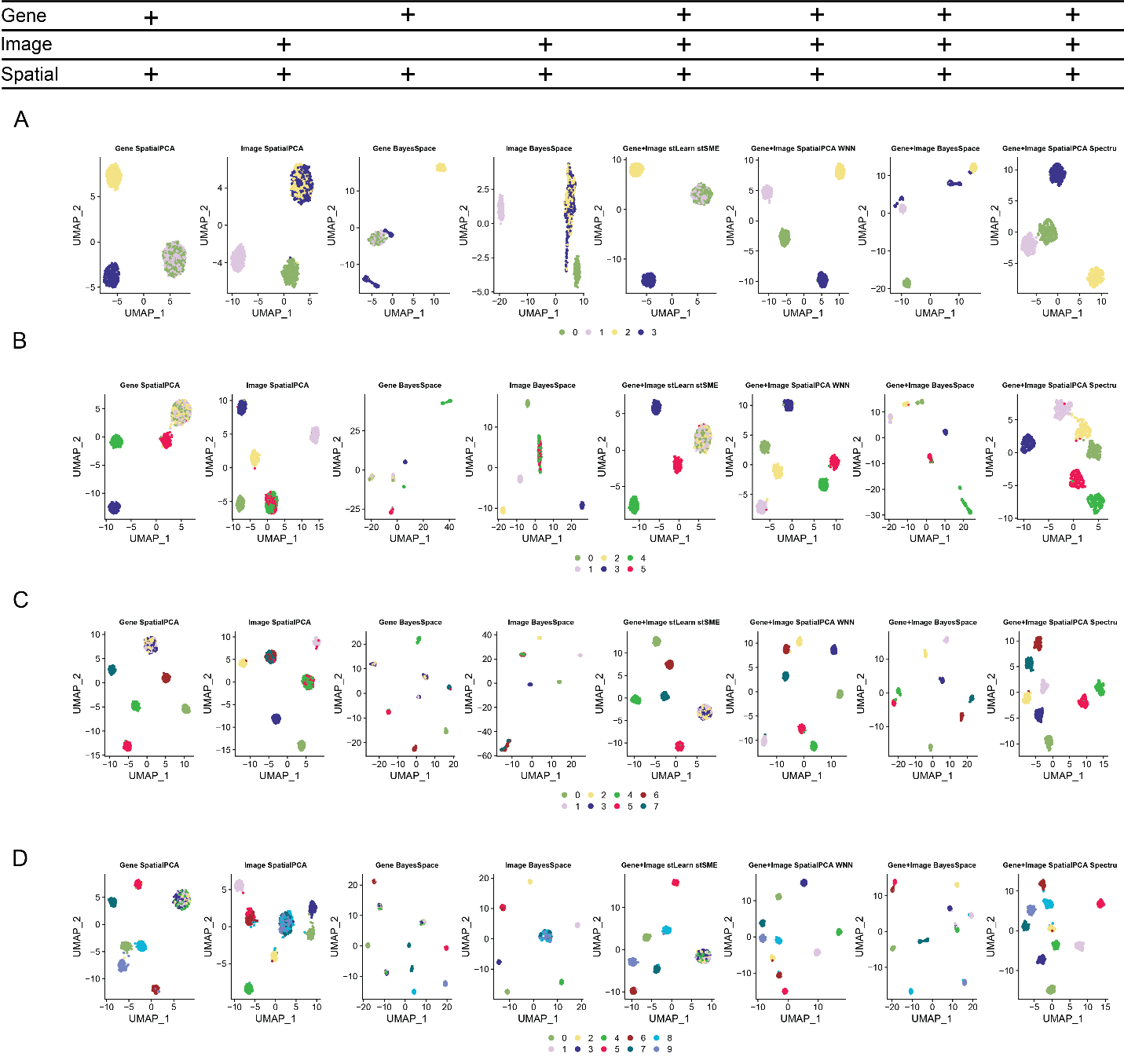


**Supplementary Figure 4:** UMAP of Simulation data with ambiguous spatial pattern.

A. Number of clusters 4; B. Number of clusters 6; C. Number of clusters 8; D. Number of clusters 10;


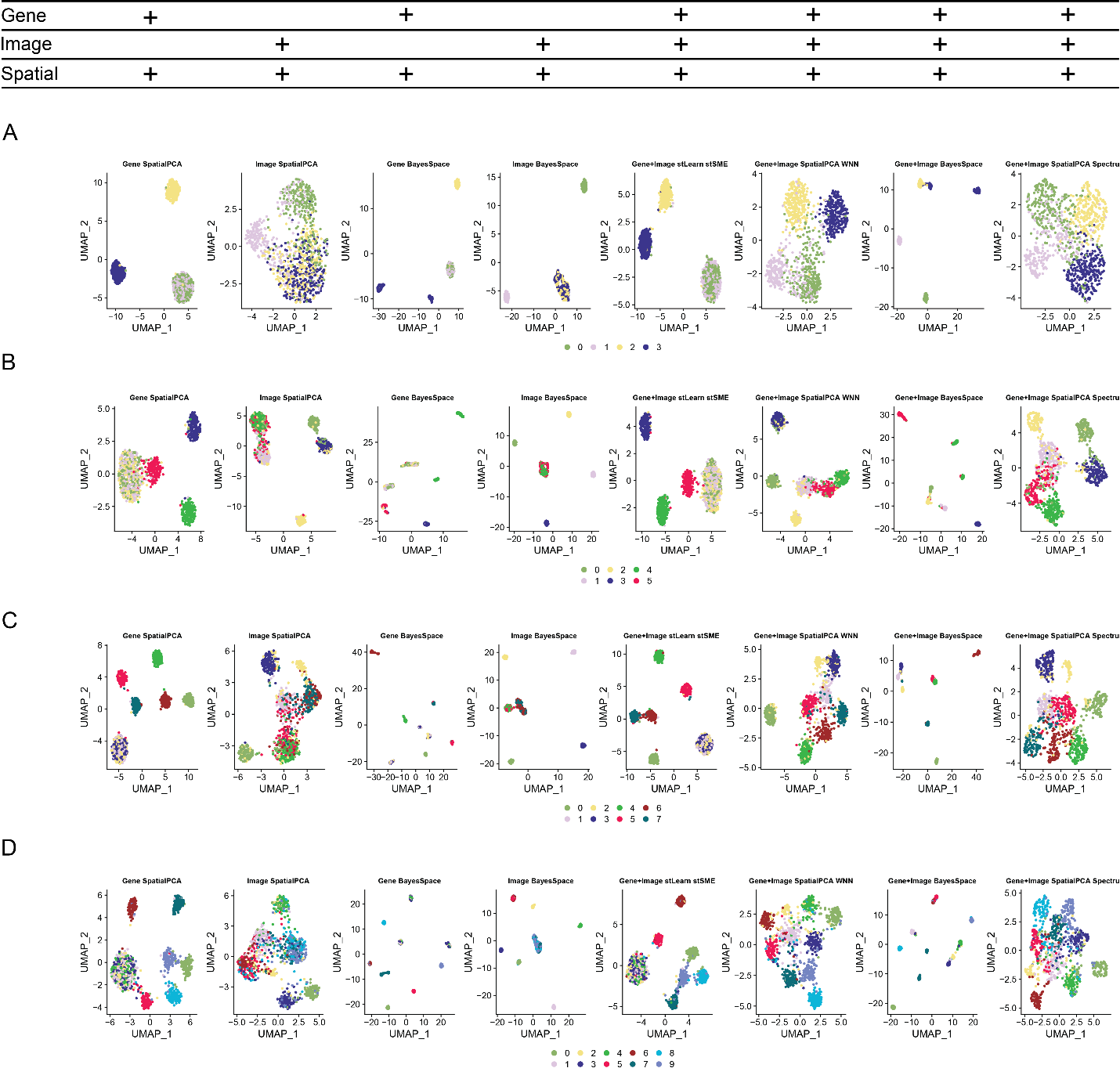


**Supplemental Figure 5**. Performance of SpaceFlow and Giotto in simulation data (Number of Clusters=10) with ambiguous (Left) and distinct (Right) spatial patterns.


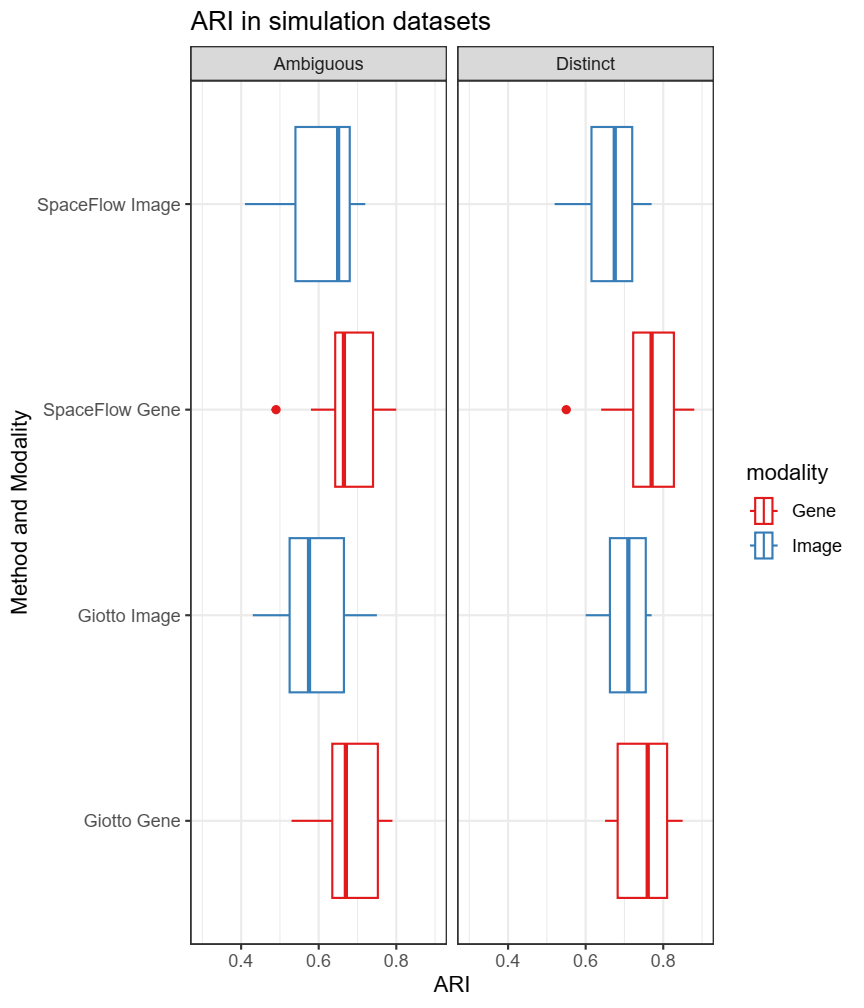


**Supplemental Figure 6**. (A) Annotated Haematoxylin and eosin (H&E) staining image from the original study (Andersson, et al., 2021). The annotations indicate regions including cancer in situ (orange), invasive cancer (red), immune infiltrate (yellow), adipose tissue (cyan), connective/fibrous tissue (blue), and normal breast glands (green). (B) Spatial region clustering results with different strategies. From left to right, clustering based on, Image Feature matrix processed by PCA (Image, plot 1), Image feature matrix processed by SpatialPCA (Image SpatialPCA, plot 2), Gene and image feature modalities processed by PCA and then integrated by TICA (TICA, plot 3), Spectrum (Spectrum, plot 4), Gene and image feature modalities processed by SpatialPCA and then integrated by Spectrum (SpatialPCA + Spectrum, plot 5), and weighted nearest neighbors of Gene and Image feature modalities processed by BayesSpace (BayesSpace + WNN, plot 6). (C) Box plot of marker genes for each cluster. (D) Enrichment analysis of differential genes between tumor and normal regions.


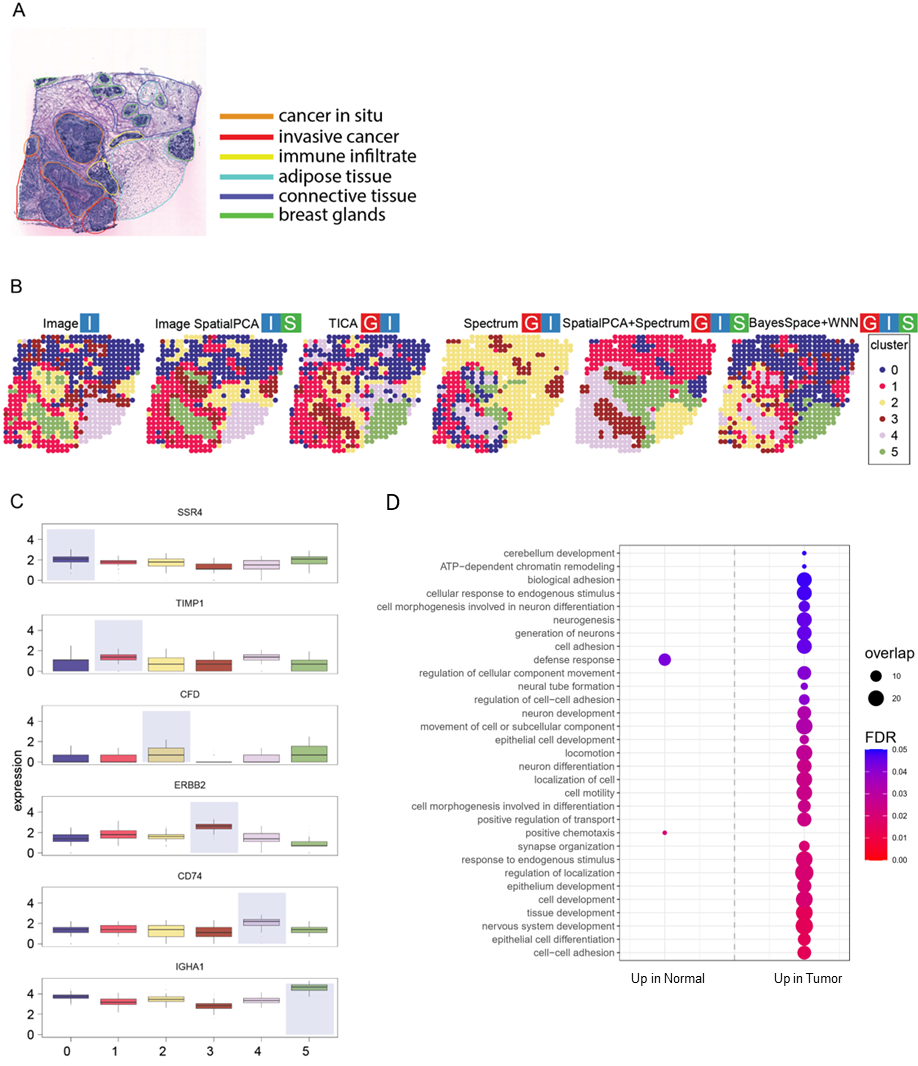


**Supplementary Figure 7:** (A) The Haematoxylin and eosin (H&E) staining image of sample PDAC-A with distinct tissue regions annotated by a pathologist from the original study. Red: cancer cells and desmoplasia; yellow: duct epithelium; blue: normal pancreatic tissue. (Moncada, et al., 2020). (B) Spatial region clustering results with different strategies. From left to right, first row, clustering based on, Gene matrix processed by PCA (Gene, plot 1), Gene matrix processed by BayesSpace (Gene BayesSpace, plot 2), Gene matrix processed by SpatialPCA (Gene SpatialPCA, plot 3), weighted nearest neighbors of Gene and Image feature modalities processed by PCA (WNN, plot4), and Gene matrix processed by stLearn (stLearn, plot 5); second row, clustering based on, Image Feature matrix processed by PCA (Image, plot 6), Image feature matrix processed by BayesSpace (Image BayesSpace, plot 7), Image feature matrix processed by SpatialPCA (Image SpatialPCA, plot 8), Gene and image feature modalities processed by BayesSpace and then integrated by WNN (BayesSpace + WNN, plot 9), and Gene and image feature modalities processed by SpatialPCA and then integrated by Spectrum (SpatialPCA + Spectrum, plot 10).


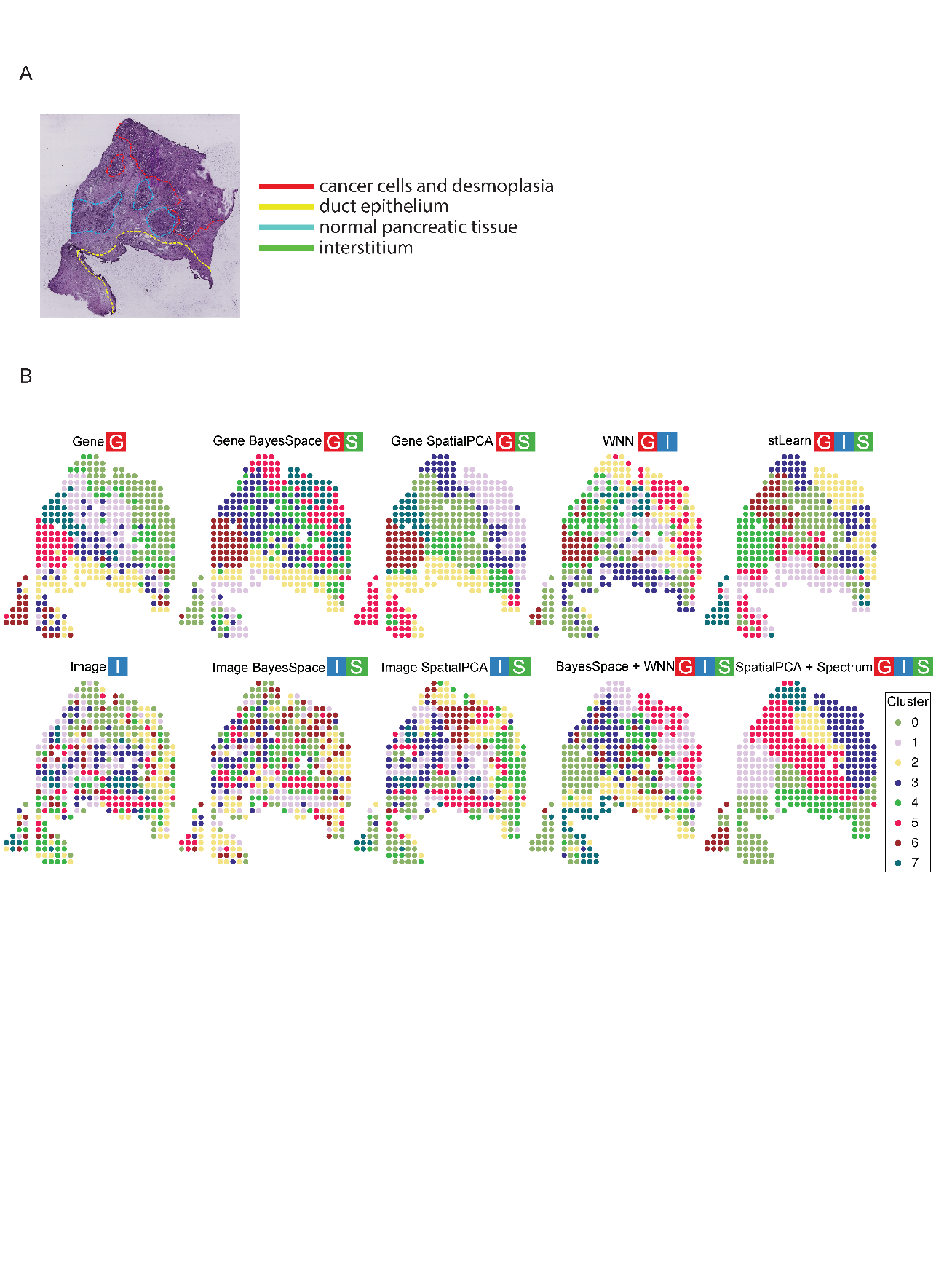


**Supplementary Figure 8:** Histology image and clustering results by other methods in sample 151673. A. The histology image showed a different pattern in image than the known layers in DLPFC samples; B. Clustering results by image PCA and other modality integration methods were dominated by patterns from image modality.


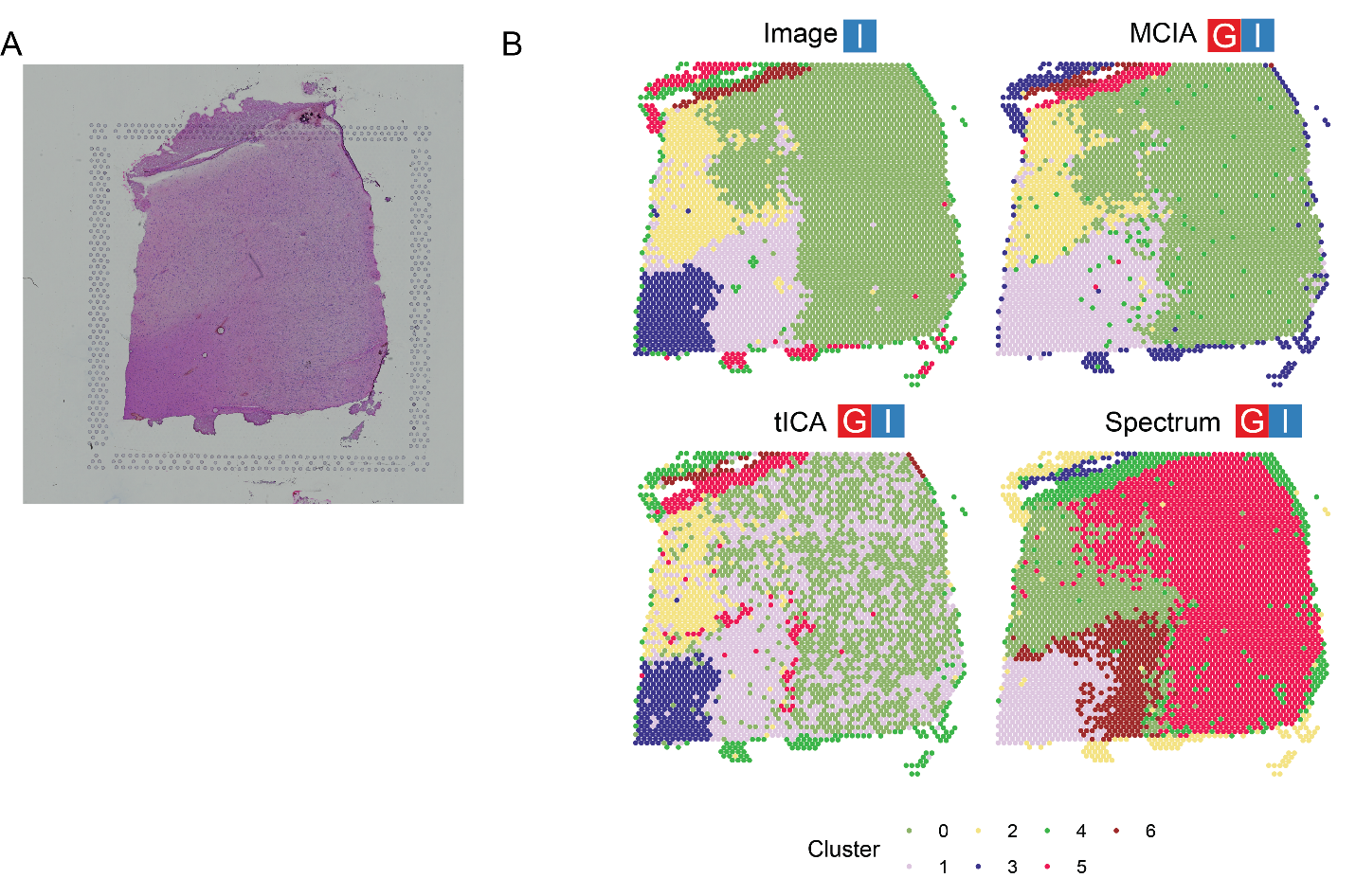


**Supplementary Figure 9:** Adjusted Rand Index (ARI) of clustering results in 12 DLPFC samples;

A. ARI in methods mentioned in Figure 5. Methods refining gene expression using local image similarity (stLearn or stLearn plus another method) improved the performance; B. Clustering by other methods showed lower ARIs;


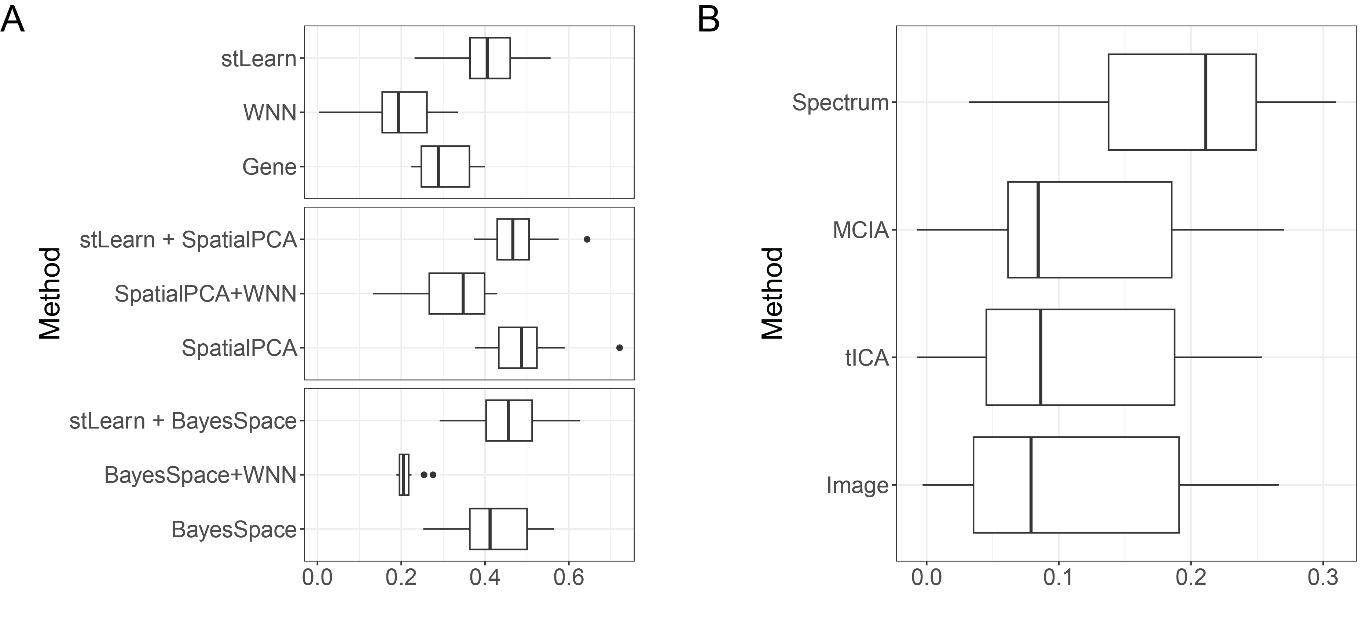


**Supplementary Figure 10:** Spatial expression pattern of marker genes from different methods. A. Marker genes from SpatialPCA cluster 1 (Top) and cluster 5 (Bottom). Both clusters were in the inner stripe region, and their cluster-specific marker genes were expressed similarly in these two clusters. B. Marker genes from SpatialPCA cluster 3 (Top) and cluster 4(Bottom). Cluster 3 were in inner medulla region while Cluster 4 contained part of inner medulla region and connective tissues. Marker genes from Cluster 3 were expressed in Cluster 4 too. While marker genes from Cluster 4 were either only expressed in connective tissues or expressed in both Cluster 3 and Cluster 4. C. Marker genes from WNN cluster 1. This cluster contained the entire inner stripe region and the marker genes were inner stripe-specific markers. D. Marker genes from WNN cluster 3. This cluster contained the entire inner medulla region and the marker genes were inner medulla-specific markers.


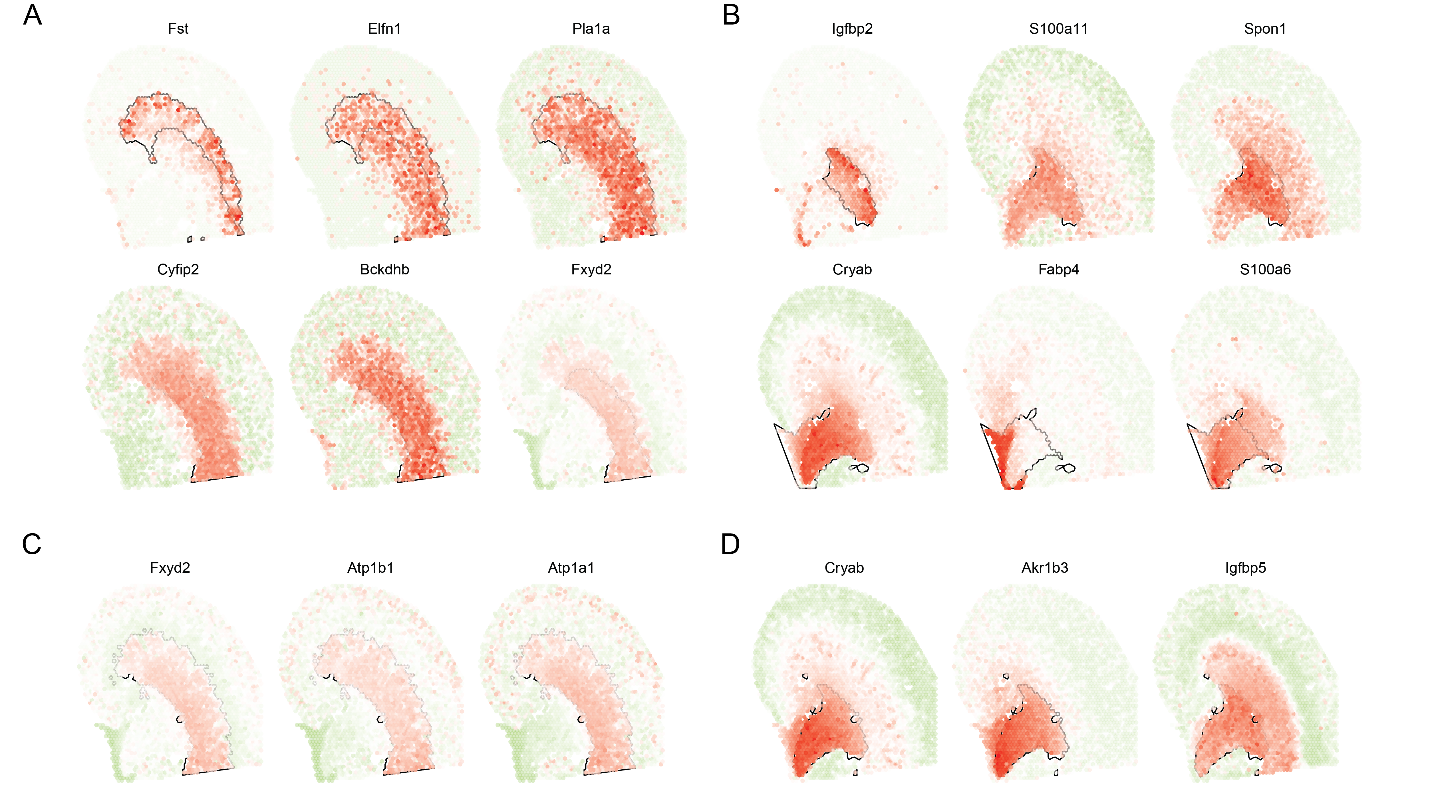


**Supplementary Figure 11: Diagnostic Plots for Evaluating Spatially Aware and Modality Integration Assumptions in simulation data.** (A) Scatterplots of Euclidean distances in the gene modality (Y-axis) versus the image modality (X-axis) for each spot pair, computed using the top 20 principal components from each modality. Each point represents a pair of spots. (B) Histograms comparing the mean similarity of each spot to its spatial neighbors versus randomly selected non-neighbors. Higher neighbor similarity (blue bars) supports the spatially aware processing assumption; conversely, elevated non-neighbor similarity (red bars) indicates possible violations of that assumption. (C) Scatterplots illustrating spatial heterogeneity by comparing each spot’s similarity to its most similar neighbor (or all neighbors) against its most similar non-neighbor. Spots marked in red highlight instances where a non-neighbor is more similar than a neighbor.


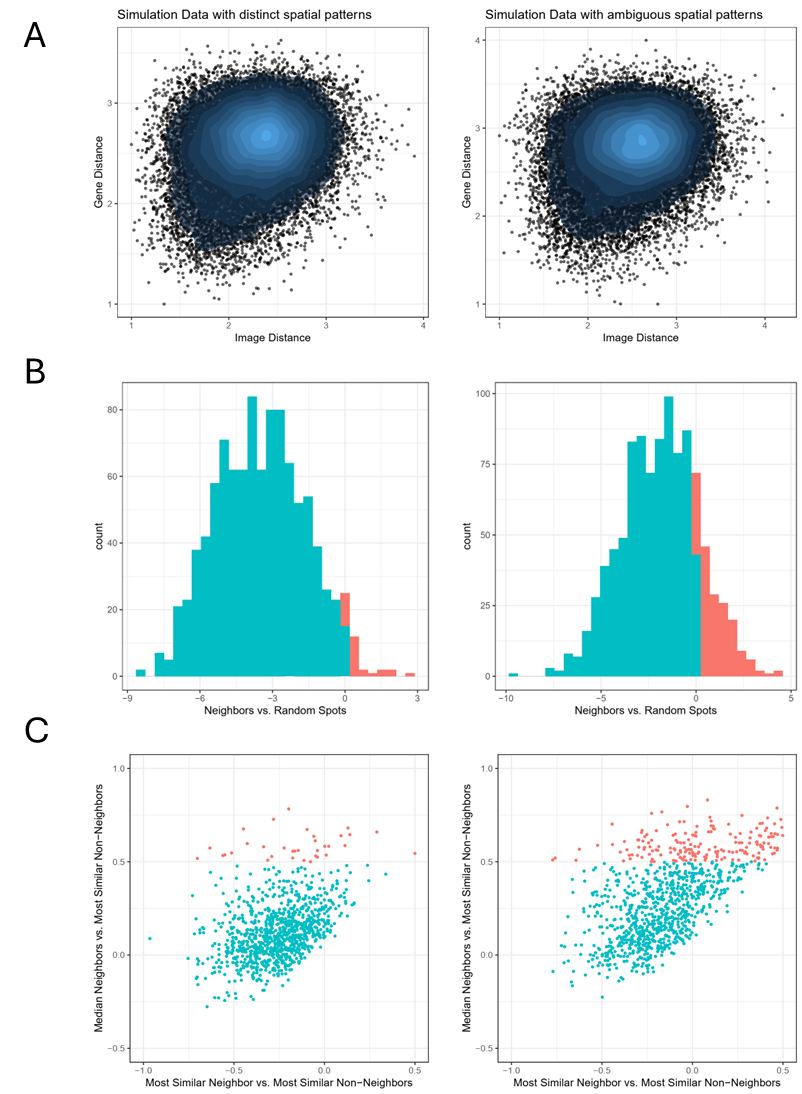


**Supplementary Figure 12: stImage analysis results in mouse brain Visium HD data**

A. Diagnostic Plots. B. Clustering results from the strategy PCA+WNN (Left) and Gene expression PCA only (Right). C. Hippocampus clusters from the two strategies, CA: Cornu Ammonis, DG: Dentate Gyrus. D. CA marker (Prkcb) genes expression in hippocampus region. and DG marker (Slc4a4) genes expression in hippocampus region.


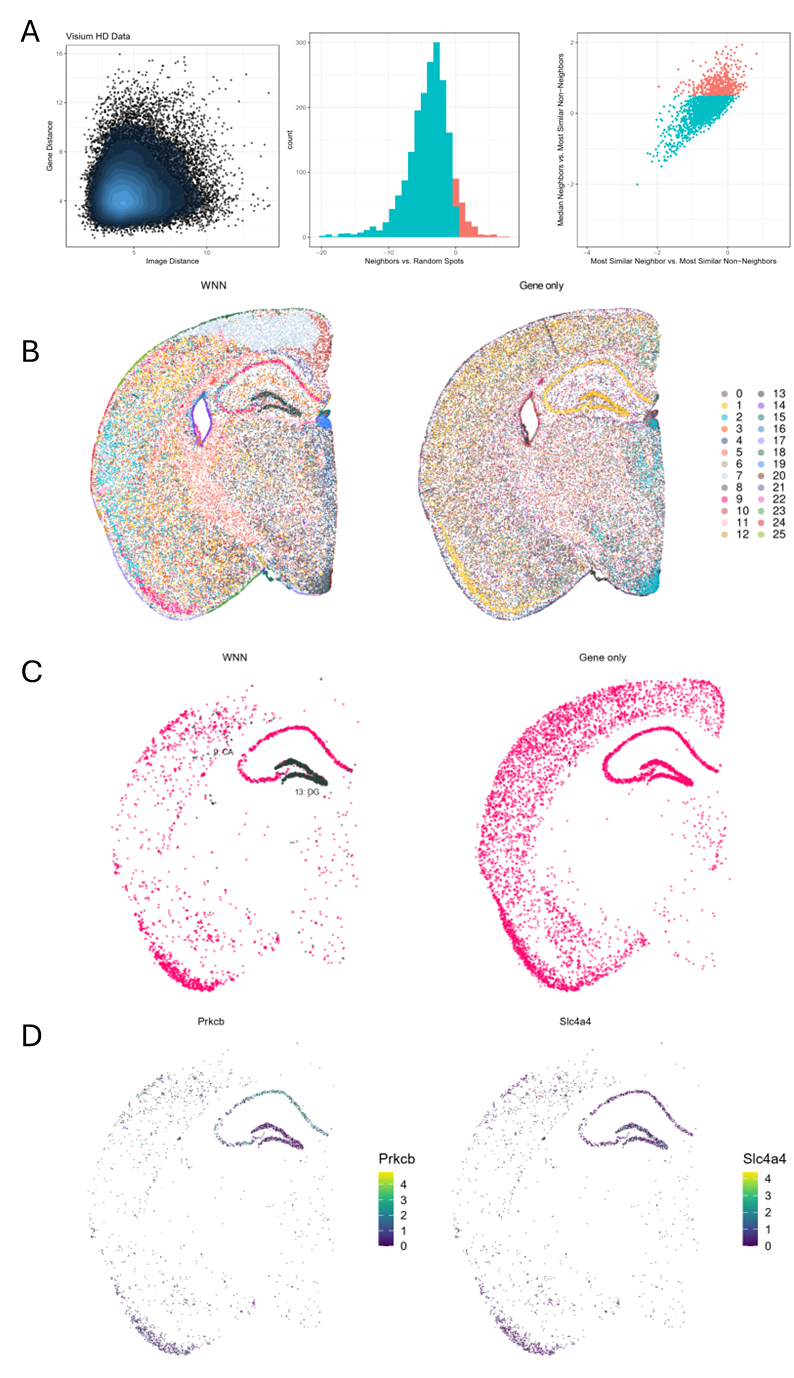


**Supplementary Figure 13: Guidance for utilizing the diagnostic plots**

**
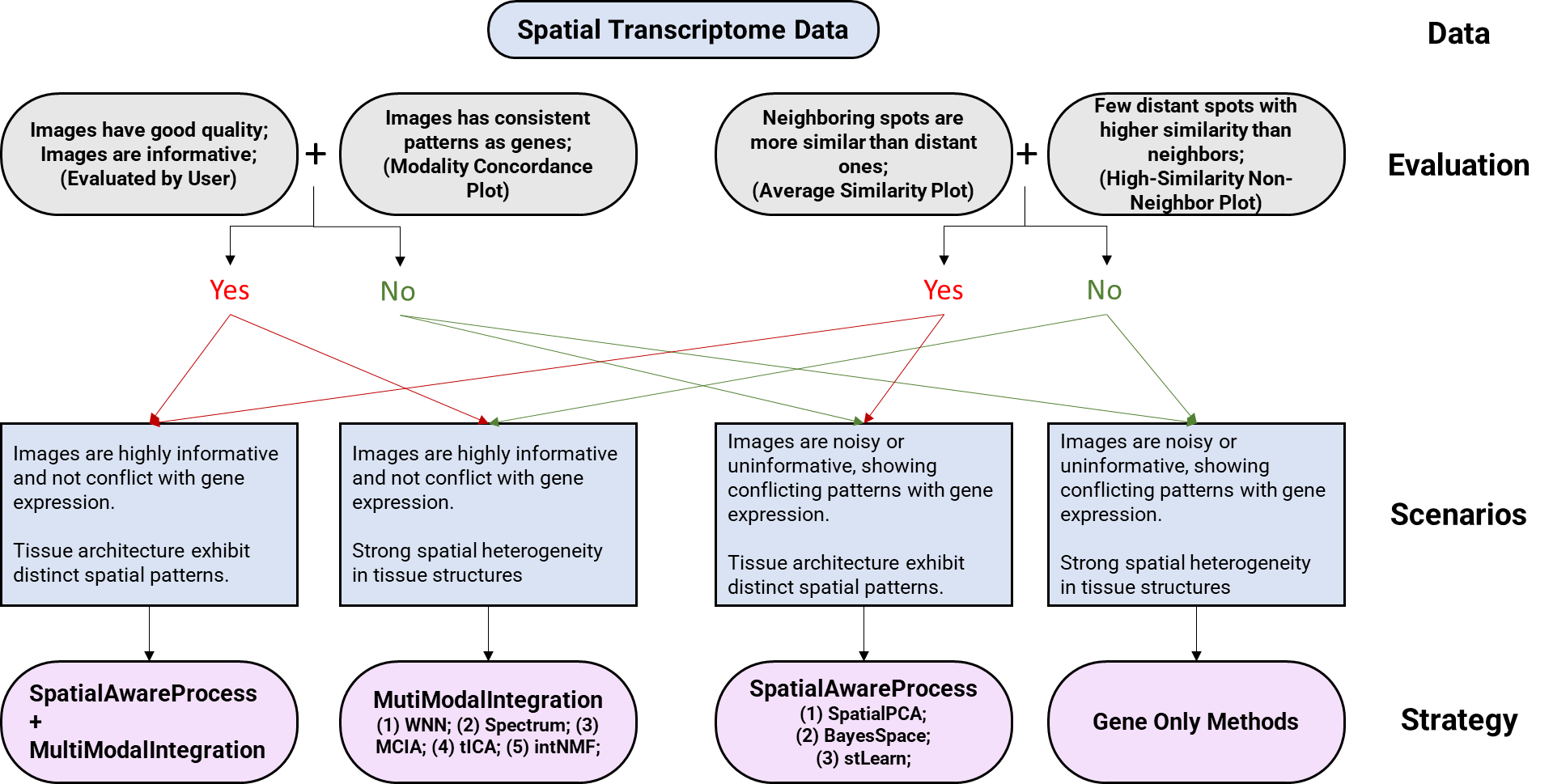
**

**Supplemental Table 1**. All strategies of processing and integrating modalities of ST data provided by stImage. Each row represents a strategy of processing ST data by combining different modalities, and preprocesing, spatially aware integration, and multi-modalities integration methods. The methods were color-matched with corresponding modalities.

**Supplemental Table 2**. Benchmark Runtime and Resource Usage of stImage Across Multiple Datasets. We evaluated the runtime performance of stImage using a typical desktop setup (CPU: Intel i7-12700; GPU: NVIDIA RTX 3060 Ti; RAM: 32 GB) for representative workflows on ST, Visium, and Visium HD datasets.

| Data set | Platform | Size | Methods/Steps | Running Time |
| --- | --- | --- | --- | --- |
| HER2 | ST | 613 spots | Image Feature Extraction | 11 Minutes |
| HER2 | ST | 613 spots * 15,029 Genes | MutiModal  Integration by WNN | 2 Minutes |
| HER2 | ST | 613 spots * 15,029 Genes | SpatialAwareProcess by spatialPCA | 7 Mintes |
| DLPFC | Visium | 3,639 spots | Image Feature Extraction | 33 Minutes |
| DLPFC | Visium | 3,639 spots * 33538 Genes | MutiModal  Integration by WNN | 5 Minutes |
| DLPFC | Visium | 3,639 spots * 33538 Genes | SpatialAwareProcess by spatialPCA | 10 Mintes |
| Mouse Brain | VisiumHD | 50k bins | Image Feature Extraction | 112 Minutes |
| Mouse Brain | VisiumHD | 50k bins * 19,059 Genes | MutiModal  Integration by WNN | 190 Minutes |
| Mouse Brain | VisiumHD | 50k bins * 19,059 Genes | SpatialAwareProcess by spatialPCA | Error. Not Support HD data |
